# Supplementary material for: Changes in risk habits and influencing factors in the Taiwan oral cancer screening program
Source: PLoS One. 2025 Jun 18;20(6):e0320461. doi: 10.1371/journal.pone.0320461 (PMC12176207; doi:10.1371/journal.pone.0320461)
Supplement: S1 File — S1 Table Characteristics of oral habit change used in the logistic regression model (improve and not improve). S2 Table The number of study participants per number of visits. (ZIP) [file pone.0320461.s001.zip › S1 Table.docx]

**S1 Table Characteristics of oral habit change used in the logistic regression model (improve and not improve)**

| **Oral habit change** | | **Variables related to oral habit changes** | | | | | | | | | | | |
| --- | --- | --- | --- | --- | --- | --- | --- | --- | --- | --- | --- | --- | --- |
|  |  | **Male (1,585,579)** | | | **Female (210,463)** | | | **Positive OPMDs (163,659)** | | | **Negative OPMDs (1,632,383)** | | |
|  |  | **Total** | **Improve (%)** | **Not improve (%)** | **Total** | **Improve (%)** | **Not improve (%)** | **Total** | **Improve (%)** | **Not improve (%)** | **Total** | **Improve (%)** | **Not improve (%)** |
| NB | MS | 442,646 | 7.7 | 92.3 | 105,361 | 13.2 | 86.8 | 21,353 | 6.8 | 93.2 | 526,654 | 8.8 | 91.2 |
|  | HS | 259,675 | 31.1 | 68.9 | 34,573 | 47.7 | 52.3 | 19,388 | 28 | 72 | 274,860 | 33.5 | 66.5 |
| QB | MS | 184,488 | 23.9 | 76.1 | 10,638 | 26.3 | 73.7 | 17,271 | 20.1 | 79.9 | 177,855 | 24.5 | 75.5 |
|  | HS | 154,744 | 45.8 | 54.2 | 4,700 | 54.4 | 45.6 | 22,847 | 43.2 | 56.8 | 136,597 | 46.6 | 53.4 |
| LB | LS | 85,692 | 33.3 | 66.7 | 16,447 | 40 | 60 | 6,482 | 25.2 | 74.8 | 95,657 | 35 | 65 |
|  | MS | 56,209 | 52.2 | 47.8 | 3,473 | 62.8 | 37.2 | 5,923 | 46.5 | 53.5 | 53,759 | 53.5 | 46.5 |
|  | HS | 43,734 | 69.7 | 30.3 | 1,828 | **75.4** | 24.6 | 6,313 | 68.2 | 31.8 | 39,249 | **70.2** | 29.8 |
| MB | LS | 38,919 | 43.2 | 56.8 | 14,081 | 39.5 | 60.5 | 5,203 | 36.8 | 63.2 | 47,797 | 42.9 | 57.1 |
|  | MS | 106,249 | 52.9 | 47.1 | 7,101 | 60.6 | 39.4 | 13,222 | 46.3 | 53.7 | 100,128 | 54.3 | 45.7 |
|  | HS | 85,113 | **74.1** | 25.9 | 2,506 | **78.9** | 21.1 | 14,184 | **71.6** | 28.4 | 73,435 | **74.7** | 25.3 |
| HB | LS | 16,449 | 46.4 | 53.6 | 6,139 | 55 | 45 | 4,082 | 45.6 | 54.4 | 18,506 | 49.4 | 50.6 |
|  | MS | 18,498 | 55 | 45 | 1,327 | 69.1 | 30.9 | 4,398 | 54.5 | 45.5 | 15,427 | 56.4 | 43.6 |
|  | HS | 93,163 | **72.5** | 27.5 | 2,289 | **78.7** | 21.3 | 22,993 | **70.6** | 29.4 | 72,459 | **73.3** | 26.7 |

Oral habits: NB-never chewing betel quid, QB-quit the chewing, LB-low degree chewing, MB-medium degree chewing, HB-high degree chewing, LS-never or quit or low degree smoking, MS-medium degree smoking, and HS-high degree smoking.

**S1 Table Characteristics of oral habit change used in the logistic regression model (improve and not improve) (continue)**

| **Oral habit change** | **Variables related to oral habit changes** | | | | | | | | |
| --- | --- | --- | --- | --- | --- | --- | --- | --- | --- |
|  | **30-45 years old (838,179)** | | | **46-60 years old (665,741)** | | | **61+ years old (292,122)** | | |
|  | **Total** | **Improve (%)** | **Not improve (%)** | **Total** | **Improve (%)** | **Not improve (%)** | **Total** | **Improve (%)** | **Not improve (%)** |
| NBMS | 273,923 | 9.6 | 90.4 | 182,137 | 7.9 | 92.1 | 91,947 | 8.1 | 91.9 |
| NBHS | 118,853 | 35.1 | 64.9 | 120,970 | 31 | 69 | 54,425 | 33.3 | 66.7 |
| QBMS | 87,822 | 17.7 | 82.3 | 71,635 | 24.9 | 75.1 | 35,669 | 38.2 | 61.8 |
| QBHS | 60,773 | 41.9 | 58.1 | 72,986 | 45.5 | 54.5 | 25,685 | 57.7 | 42.3 |
| LBLS | 48,467 | 24.7 | 75.3 | 34,265 | 37.7 | 62.3 | 19,407 | 52.7 | 47.3 |
| LBMS | 38,768 | 48.1 | 51.9 | 15,904 | 58.4 | 41.6 | 5,010 | **71.8** | 28.2 |
| LBHS | 28,735 | 65.8 | 34.2 | 13,874 | **75.4** | 24.6 | 2,953 | **83.8** | 16.2 |
| MBLS | 15,588 | 34.1 | 65.9 | 22,283 | 43 | 57 | 15,129 | 49.6 | 50.4 |
| MBMS | 55,519 | 47.5 | 52.5 | 41,329 | 54.7 | 45.3 | 16,502 | 69.7 | 30.3 |
| MBHS | 46,308 | **70.9** | 29.1 | 33,512 | **76.6** | 23.4 | 7,799 | **83.8** | 16.2 |
| HBLS | 5,974 | 37.7 | 62.3 | 10,315 | 48.7 | 51.3 | 6,299 | 59.2 | 40.8 |
| HBMS | 10,318 | 51.3 | 48.7 | 7,271 | 57.3 | 42.7 | 2,236 | **73** | 27 |
| HBHS | 47,131 | 68.9 | 31.1 | 39,260 | **74.3** | 25.7 | 9,061 | **85** | 15 |

Oral habits: NB-never chewing betel quid, QB-quit the chewing, LB-low degree chewing, MB-medium degree chewing, HB-high degree chewing, LS-never or quit or low degree smoking, MS-medium degree smoking, and HS-high degree smoking.

**S1 Table Characteristics of oral habit change used in the logistic regression model (improve and not improve) (continue)**

| **Oral habit change** | **Variables related to oral habit changes** | | | | | | | | | | | |
| --- | --- | --- | --- | --- | --- | --- | --- | --- | --- | --- | --- | --- |
|  | **Elementary School (270,656)** | | | **Middle and High school (849,639)** | | | **College+ (323,008)** | | | **Unknown (352,739)** | | |
|  | **Total** | **Improve (%)** | **Not improve (%)** | **Total** | **Improve (%)** | **Not improve (%)** | **Total** | **Improve (%)** | **Not improve (%)** | **Total** | **Improve (%)** | **Not improve (%)** |
| NBMS | 65,787 | 6.3 | 93.7 | 225,990 | 7.9 | 92.1 | 144,485 | 10.1 | 89.9 | 111,745 | 10.2 | 89.8 |
| NBHS | 43,581 | 29.7 | 70.3 | 135,234 | 30.5 | 69.5 | 57,342 | 40.3 | 59.7 | 58,091 | 34.6 | 65.4 |
| QBMS | 30,520 | 31.7 | 68.3 | 95,390 | 21.3 | 78.7 | 32,544 | 21.6 | 78.4 | 36,672 | 27.2 | 72.8 |
| QBHS | 26,807 | 51 | 49 | 84,382 | 43.3 | 56.7 | 19,567 | 48.7 | 51.3 | 28,688 | 47.8 | 52.2 |
| LBLS | 18,035 | 40.8 | 59.2 | 46,340 | 28.7 | 71.3 | 14,432 | 33.6 | 66.4 | 23,332 | 41.3 | 58.7 |
| LBMS | 6,229 | 58.8 | 41.2 | 30,654 | 48.6 | 51.4 | 11,562 | 57.3 | 42.7 | 11,237 | 56.4 | 43.6 |
| LBHS | 4,600 | **75.8** | 24.2 | 24,727 | 67.4 | 32.6 | 7,760 | **73.5** | 26.5 | 8,475 | **70.6** | 29.4 |
| MBLS | 15,914 | 43.2 | 56.8 | 21,850 | 39 | 61 | 4,307 | 41.5 | 58.5 | 10,929 | 47.7 | 52.3 |
| MBMS | 19,223 | 59.6 | 40.4 | 59,418 | 48.8 | 51.2 | 12,716 | 59.7 | 40.3 | 21,993 | 56.4 | 43.6 |
| MBHS | 12,545 | **77.8** | 22.2 | 50,083 | **72.5** | 27.5 | 9,617 | **78.4** | 21.6 | 15,374 | **74.3** | 25.7 |
| HBLS | 7,751 | 54.7 | 45.3 | 9,219 | 43.3 | 56.7 | 1,149 | 43.6 | 56.4 | 4,469 | 50.9 | 49.1 |
| HBMS | 3,563 | 63 | 37 | 11,118 | 52.3 | 47.7 | 1,340 | 59.3 | 40.7 | 3,804 | 58.9 | 41.1 |
| HBHS | 16,101 | **76.7** | 23.3 | 55,234 | **70.7** | 29.3 | 6,187 | **77.2** | 22.8 | 17,930 | **73.4** | 26.6 |

Oral habits: NB-never chewing betel quid, QB-quit the chewing, LB-low degree chewing, MB-medium degree chewing, HB-high degree chewing, LS-never or quit or low degree smoking, MS-medium degree smoking, and HS-high degree smoking.

**S1 Table Characteristics of oral habit change used in the logistic regression model (improve and not improve) (continue)**

| **Oral habit change** | **Variables related to oral habit changes** | | | | | |
| --- | --- | --- | --- | --- | --- | --- |
|  | **Screening 2 times (850,005)** | | | **Screening more than 2 times (946,037)** | | |
|  | **Total** | **Improve (%)** | **Not improve (%)** | **Total** | **Improve (%)** | **Not improve (%)** |
| NBMS | 280,421 | 10.4 | 89.6 | 267,586 | 7.1 | 92.9 |
| NBHS | 135,042 | 33.3 | 66.7 | 159,206 | 32.9 | 67.1 |
| QBMS | 95,211 | 25.2 | 74.8 | 99,915 | 23 | 77 |
| QBHS | 72,704 | 44 | 56 | 86,740 | 47.9 | 52.1 |
| LBLS | 49,186 | 37.6 | 62.4 | 52,953 | 31.4 | 68.6 |
| LBMS | 27,441 | 52.1 | 47.9 | 32,241 | 53.5 | 46.5 |
| LBHS | 19,729 | 67.8 | 32.2 | 25,833 | **71.5** | 28.5 |
| MBLS | 22,892 | 43.9 | 56.1 | 30,108 | 41 | 59 |
| MBMS | 50,261 | 52.3 | 47.7 | 63,089 | 54.2 | 45.8 |
| MBHS | 37,681 | **71.6** | 28.4 | 49,938 | **76.2** | 23.8 |
| HBLS | 8,901 | 47.2 | 52.8 | 13,687 | 49.7 | 50.3 |
| HBMS | 8,831 | 53.7 | 46.3 | 10,994 | 57.8 | 42.2 |
| HBHS | 41,705 | 69.4 | 30.6 | 53,747 | **75.2** | 24.8 |

Oral habits: NB-never chewing betel quid, QB-quit the chewing, LB-low degree chewing, MB-medium degree chewing, HB-high degree chewing, LS-never or quit or low degree smoking, MS-medium degree smoking, and HS-high degree smoking.
