# Supplementary material for: Changes in risk habits and influencing factors in the Taiwan oral cancer screening program
Source: PLoS One. 2025 Jun 18;20(6):e0320461. doi: 10.1371/journal.pone.0320461 (PMC12176207; doi:10.1371/journal.pone.0320461)
Supplement: S1 File — S1 Table Characteristics of oral habit change used in the logistic regression model (improve and not improve). S2 Table The number of study participants per number of visits. (ZIP) [file pone.0320461.s001.zip › S2 Table.docx]

**S2 Table The number of study participants per number of visits**

| Frequency of visit | Number of participants (5,423,967) | Percent |
| --- | --- | --- |
| 1 | 2854047 | 52.6 |
| 2 | 1311355 | 24.2 |
| 3 | 690821 | 12.7 |
| 4 | 353064 | 6.5 |
| 5 | 156298 | 2.9 |
| 6 | 49688 | 0.9 |
| 7 | 7801 | 0.1 |
| 8 | 817 | 0 |
| 9 | 70 | 0 |
| 10 | 6 | 0 |

A total of 5.4 million participants have joined the screening program, of which 2,569,920 individuals (47.4%) participated more than once.
