# Supplementary material for: Changes in risk habits and influencing factors in the Taiwan oral cancer screening program
Source: PLoS One. 2025 Jun 18;20(6):e0320461. doi: 10.1371/journal.pone.0320461 (PMC12176207; doi:10.1371/journal.pone.0320461)
Supplement: Attachment — Submitted filename: Response to Reviewer II.docx [file pone.0320461.s005.docx]

**Response to Reviewer II**

**Comment:** We note your reply to our data availability query as follows:

"The individual-level raw data used in our study cannot be made publicly available due to ethical and legal restrictions, as specified in the protocol approved by the Research Ethics Committee C of the National Taiwan University Hospital. Public release of these data would risk compromising participant confidentiality. However, we have provided the aggregated numbers by screening mode and participant "

PLOS journals require authors to make the minimal data set publicly available without restriction at the time of publication. PLOS defines the "minimal data set" as consisting of the data used to reach the conclusions drawn in the manuscript with related metadata and methods, and any additional data required to replicate the reported study findings in their entirety. This includes:
- The values behind the means, standard deviations and other measures reported;
- The values used to build graphs;
- The points extracted from images for analysis
Additionally, PLOS requires that authors comply with field-specific standards for preparation, recording, and deposition of data when applicable.

Can you please confirm if the aggregated numbers you note you have provided would constitute your minimal data set?

**Response:**

Thank you for your follow-up regarding data availability.

We confirm that we have uploaded the **new** aggregate-level dataset, which contains the minimal data set used to support the findings of our study, to the Dryad repository. This dataset includes all relevant values used to generate statistical results and conclusions presented in the manuscript, such as:

- Aggregated counts used to calculate proportions and rates;
- Values behind reported means or percentages;
- Data used to build tables and figures, including habit change patterns, screening frequencies, and OPMD findings across subgroups.
- The dataset is accessible via the following: <https://doi.org/10.5061/dryad.612jm64h3>

or

http://datadryad.org/share/_Y6sTNL-JPFygiwCL95Q9EZaCD_wVZBrRlSoGUC64_A

We will enter this DOI into the full Data Availability Statement in the "Additional Information" section of the PLOS submission form, as required. We will also provide the URL passcode in the "Attach Files" section.

This uploaded file constitutes our minimal data set as defined by PLOS and complies with both journal policy and the ethical requirements approved by the Research Ethics Committee of the National Taiwan University Hospital.

We add this information to the manuscript: “The aggregated dataset used in this study has been publicly uploaded to the Dryad repository and is available at: <https://doi.org/10.5061/dryad.612jm64h3>.” [line: 114-115]
